# A Single Lys Residue on the First Intracellular Loop Modulates the Endoplasmic Reticulum Export and Cell-Surface Expression of α_2A_-Adrenergic Receptor

**DOI:** 10.1371/journal.pone.0050416

**Published:** 2012-12-05

**Authors:** Yi Fan, Chunman Li, Jianhui Guo, Gang Hu, Guangyu Wu

**Affiliations:** 1 Department of Pharmacology and Toxicology, Georgia Health Sciences University, Augusta, Georgia, United States of America; 2 Department of Pharmacology, Nanjing Medical University, Nanjing, China; Beatson Institute for Cancer Research Glasgow, United Kingdom

## Abstract

Export from the endoplasmic reticulum (ER) represents an initial step in intracellular trafficking of G protein-coupled receptors (GPCRs). However, the underlying molecular mechanisms remain poorly understood. We have previously demonstrated that a highly conserved Leu residue on the first intracellular loop (ICL1) is required for exit of several GPCRs from the ER. Here we found that, in addition to Leu64 residue in the ICL1, the neighboring positively charged residue Lys65also modulates the cell-surface transport of α_2A_-adrenergic receptor (α_2A_-AR). Mutation of Lys65 to Ala, Glu and Gln significantly attenuated, whereas mutation of Lys65 to Arg strongly augmented α_2A_-AR expression at the cell surface. Consistent with the effects on the cell-surface expression of α_2A_-AR, mutation of Lys65 to Ala and Arg produced opposing effects on α_2A_-AR-mediated ERK1/2 activation. Furthermore, confocal microscopy revealed that the α_2A_-AR mutant K65A displayed a strong intracellular expression pattern and was extensively co-localized with the ER marker DsRed2-ER, suggestive of ER accumulation. These data provide the first evidence indicating an important function for a single Lys residue on the ICL1 in the ER export and cell-surface expression of α_2A_-AR. These data also suggest that the ICL1 may possess multiple signals that control the cell-surface targeting of GPCRs via distinct mechanisms.

## Introduction

G protein-coupled receptors (GPCRs) constitute the largest superfamily of cell surface receptors and regulate the cellular responses to a broad spectrum of extracellular signals, such as hormones, neurotransmitters, chemokines, proteinases, odorants, light and calcium ions [1]–[4]. All GPCRs share a common molecular topology with a hydrophobic core of seven membrane-spanning α-helices, three intracellular loops, three extracellular loops, an N-terminus outside the cell, and a C-terminus inside the cell. The proper function of GPCRs is largely determined by the highly regulated intracellular trafficking of the receptors. GPCRs are synthesized in the ER and after proper folding and correct assembly, they transport to the cell surface *en route* through the Golgi apparatus and trans-Golgi network. As the first step in post-translational biogenesis, the efficiency of ER export of nascent GPCRs plays a crucial role in the regulation of maturation, cell-surface expression, and physiological functions of the receptors [5]–[8].

Great progress has been made on the understanding of GPCR export from the ER over the past decade [5], [7]. However, the underlying molecular mechanisms remain much less-well understood as compared with extensive studies on the events involved in the endocytic and recycling pathways [9]–[14]. It has been demonstrated that, similar to many other plasma membrane proteins, GPCRs must first attain native conformation in order to exit from the ER. Incompletely or misfolded receptors are excluded from ER-derived transport vesicles by the ER quality control mechanism [15]–[17]. It is also clear that GPCR export from the ER is modulated by direct interactions with a multitude of regulatory proteins such as ER chaperones and receptor activity modifying proteins (RAMPs), which may stabilize receptor conformation, facilitate receptor maturation and promote receptor delivery to the plasma membrane [18]–[23]. More interestingly, a number of highly conserved, specific sequences or motifs embedded within the receptors have recently been indentified to dictate receptor export from the ER [24]–[33]. Although the molecular mechanisms underlying the function of these motifs remain elusive, they may modulate proper receptor folding in the ER or receptor interaction with specific components of transport machinery [5], [15], [34], [35].

There are three α_2_-AR subtypes, designated as α_2A_-AR, α_2B_-AR, and α_2C_-AR. It has been known that both α_2A_-AR and α_2B_-AR mainly express at the cell surface, whereas α_2C_-AR cell-surface expression depends on the cell types [36]. We have identified several motifs, including the F(x)_6_LL motif in the C-terminus, the RRR motif in the third intracellular loop (ICL3), and an isolated Leu residue in the ICL1, which are essential for export trafficking of α_2B_-AR [15], [34], [37]–[39]. In a continuing effort to search for the structural determinants of α_2_-AR transport, we expanded our studies to define the role of the ICL1 in the cell-surface expression of α_2A_-AR. Surprisingly we found that, in addition to Leu residue, a neighboring Lys residue specifically modulates the ER export and cell-surface expression of α_2A_-AR and this function is likely dictated by its positively charged property. These data provide the first evidence indicating that the ICL1 may possess multiple signals that use distinct mechanisms to control the processing of α_2A_-AR.

## Materials and Methods

### Materials

Antibodies against ERK1/2 and phospho-ERK1/2 were from Cell Signaling Technology (Beverly, MA). The ER marker pDsRed2-ER was purchased from BD Biosciences (Palo Alto, LA). Prolong antifade reagent with DAPI was obtained from Invitrogen Life Technologies (Carlsbad, CA). UK14,304 was from Sigma (St. Louis, MO). [^3^H]-RX821002 (specific activity = 50 Ci/mmol) was from Perkin Elmer Life Sciences. All other materials were obtained as described previously [38], [40], [41].

### Plasmid Constructions

Rat α_2B_-AR in vector pcDNA3 was kindly provided by Dr. Stephen M. Lanier (Medical University of South Carolina, Charleston, SC). Human α_2A_-AR tagged with three HA at its N-terminus was purchased from UMR cDNA Resource Center (Rolla, MO). α_2A_-AR tagged with GFP at its C-terminus was generated as described previously [40]. The GFP and HA epitopes have been used to label GPCRs resulting in receptors with similar characteristics to the wild-type receptors [40], [42], [43]. The mutations of α_2A_-AR and α_2B_-AR were created by using the QuikChange site-directed mutagenesis kit (Stratagene, La Jolla, CA). The sequence of each construct used in this study was verified by restriction mapping and nucleotide sequence analysis.

### Cell Culture and Transient Transfection

HEK293 and HeLa cells were cultured in Dulbecco’s modified Eagle’s medium (DMEM) with 10% fetal bovine serum (FBS), 100 units/ml penicillin, and 100 units/ml streptomycin and transiently transfected by using Lipofectamine 2000 reagent as described previously [44]. Transfection efficiency was estimated to be greater than 70% based on the GFP fluorescence.

### Intact Cell Ligand Binding

Cell-surface expression of α_2A_-AR and α_2B_-AR in HEK293 cells was measured by ligand binding of intact live cells using the membrane impermeable ligands [^3^H]-RX821002 as described previously [41], [45]. Briefly, HEK293 cells cultured on 6-well dishes were transiently transfected with 1 µg of plasmids. After 6 h the cells were split into 24-well dishes pre-coated with poly-L-lysine. Forty-eight h post-transfection, the cells were incubated with DMEM plus [^3^H]-RX821002 in a total of 300 µl for 60 min at room temperature. In the initial experiments, the cells were incubated with increasing concentrations of [^3^H]-RX821002 (from 0.3125 to 20 nM) to generate ligand dose-dependent binding curves. Because ligand binding to the receptors is almost saturated at a concentration of 20 nM, this concentration was then used to measure the cell-surface expression of the receptors. The binding was terminated and excess radioligand eliminated by washing the cells twice with ice-cold DMEM. The retained radioligand was extracted by digesting the cells in 1 M NaOH for 2 h at room temperature. The liquid phase was collected and suspended in 4 ml of Ecoscint A scintillation fluid (National Diagnostics Inc., Atlanta, GA). The amount of radioactivity retained was measured by liquid scintillation spectrometry. The non-specific binding of α_2_-AR was determined in the presence of rauwolscine (10 µM). All radioligand binding assays were performed in triplicate.

### Flow Cytometry

For measurement of total receptor expression, HEK293 cells cultured on 6-well dishes were transiently transfected with 1 µg of GFP-tagged receptors for 24 h. The cells were collected, washed twice with PBS and re-suspended at a density of 8×10^6^ cells/ml. Total GFP fluorescence was then measured on a flow cytometer (BD Biosciences FASCalibur) as described previously (38). For measurement of the cell-surface expression of α_2A_-AR, HEK293 cells were cultured on 6-well dishes and transfected with 1 µg of HA-tagged α_2A_-AR for 24 h. The cells were then collected, suspended in PBS containing 1% FBS and incubated with high affinity anti-HA-fluorescein (3F10) at a final concentration of 2 µg/ml at 4°C for 60 min. After washing for 2×0.5 ml PBS containing 1% FBS, the cells were re-suspended and the fluorescence was analyzed as described above.

### Confocal Fluorescence Microscopy

For fluorescence microscopic analysis of receptor subcellular distribution, HEK293 and HeLa cells were grown on coverslips pre-coated with poly-L-lysine in 6-well plates and transfected with 100 ng of GFP-tagged receptors. For co-localization of GFP-tagged receptors with the ER marker DsRed2-ER, HEK293 cells grown on coverslips were transfected with 100 ng of GFP-tagged receptors and 100 ng of pDsRed2-ER. The cells were fixed with 4% paraformaldehyde-4% sucrose mixture in PBS for 15 min and the coverslips were mounted with prolong antifade reagent containing DAPI. Images were captured using a Zessis confocal microscope (LSM510) equipped with a 63x objective (NA = 1.3). The colocalization of the receptor with the ER marker DsRed2-ER was determined by Pearson’s coefficient using the ImageJ JaCoP plug-in as described [46].

### Measurement of ERK1/2 Activation

HEK293 cells were cultured in 6-well dishes and transfected with 1 µg of α_2A_-AR or its mutants as described above. After 24 h transfection, the cells were starved for at least 3 h and then stimulated with different concentrations of UK14,304 (0.01, 0.1 and 1 µM) for 5 min. Stimulation was terminated by addition of 150 µl of ice-cold cell lysis buffer containing 50 mM Tris-HCl, pH 7.4, 150 mM NaCl, 1% Nonidet P-40, 1 mM sodium orthovanadate and protease inhibitor cocktail (Roche). After solubilizing the cells on ice for 20 min, 10 µl of total cell lysates was separated by 12% SDS-PAGE and ERK1/2 activation was determined by measuring the levels of phosphorylation of ERK1/2 with phospho-specific ERK1/2 antibodies by immunoblotting [47], [48].

### Statistical Analysis

Differences were evaluated using one-way ANOVA and post-hoc Tukey’s test, and *p*<0.05 was considered as statistically significant. Data are expressed as the mean ± S.E.

## Results

### Differential Inhibition of the Cell-surface Expression of α_2A_-AR and α_2B_-AR by Mutation of Leu and a Positively Charged Residue on the ICL1

**Figure 1:**
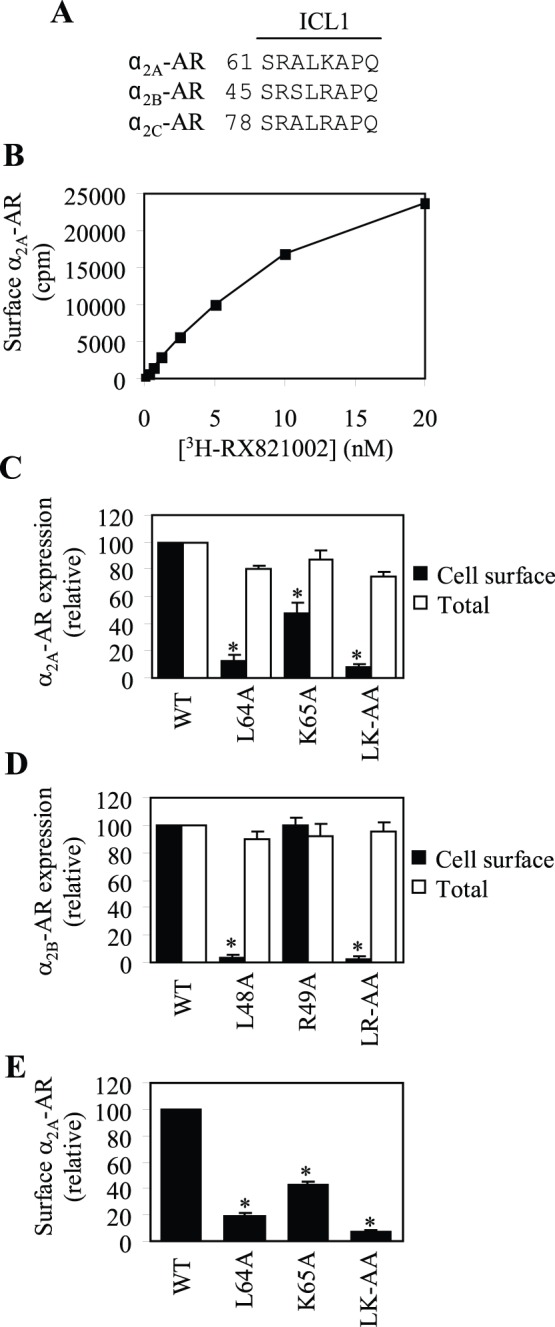
Effects of the mutation of Leu residues and their neighboring positively charged residues in the ICL1 on the cell-surface and total expression of α_2A_-AR and α_2B_-AR. (A) The sequence of the ICL1 of α_2A_-AR, α_2B_-AR and α_2C_-AR. (B) Ligand dose-dependent binding of α_2A_-AR in intact HEK293 cells. HEK293 cells cultured on 6-well plates were transfected with α_2A_-AR and then split onto 24-well plates. The cells were incubated with increasing concentrations of [^3^H]-RX821002 (0.3125, 0.625, 1.25, 2.5, 5, 10, and 20 nM) and the ligand bound to the receptor was measured by liquid scintillation spectrometry as described in the “Materials and Methods”. The nonspecific binding was determined in the presence of rauwolscine (10 µM). Similar results were obtained in at least three different experiments. (C) Quantification of the cell-surface and total expression of α_2A_-AR and its mutants in which Leu64 and Lys65 were mutated to Ala individually or in combination. (D) Quantification of the cell-surface and total expression of α_2B_-AR and its mutants in which Leu48 and Arg49 were mutated to Ala. In (C) and (D), HEK293 cells were transfected with α_2_-AR and the cell-surface expression of the receptors was measured by intact cell binding assays using [^3^H]-RX821002 at 20 nM. The mean values of specific [^3^H]-RX821002 binding were 27255±415 and 16785±452 cpm from cells transfected with wild-type (WT) α_2A_-AR and α_2B_-AR, respectively. Wild-type α_2A_-AR and α_2B_-AR and their mutants were tagged with GFP and transiently expressed in HEK293 cells. Total receptor expression was determined by flow cytometry measuring the GFP signal as described in the “Materials and Methods”. (E) Quantification of the cell-surface expression of α_2A_-AR and its mutants by flow cytometry. HEK293 cells were transfected with HA-tagged α_2A_-AR or its individual mutant and the cell-surface expression of the receptors was measured by flow cytometry following staining with anti-HA antibodies in nonpermeabilized cells as described in the “Materials and Methods”. The data shown in (C), (D), and (E) are percentages of the mean value obtained from cells transfected with wild-type α_2_-AR and are presented as the mean ± S.E. of at least three different experiments. *, *p*<0.05 *versus* respective WT α_2_-AR.

The amino acid sequences of the ICL1 are highly conserved in each α_2_-AR subtype among different species but different between three α_2_-AR subtypes. The ICL1 of α_2A_-AR, α_2B_-AR and α_2C_-AR have the sequence of ALK, SLR and ALR, respectively (Fig. 1A). We have previously demonstrated that Leu48 residue, but not Arg49 residue, in the ICL1 is essential for the ER export and cell-surface transport of α_2B_-AR [38]. Here we determined the effect of mutating Leu64 and Lys65 on the cell-surface number of α_2A_-AR. We first measured the saturation binding of the radioligand [^3^H]-RX821002 to α_2A_-AR in intact live HEK293 cells. The ligand dose-dependently bound to α_2A_-AR and the binding was close to saturation at 20 nM (Fig. 1B).

Wild-type α_2A_-AR and its mutants L64A, K65A and LK-AA were transiently expressed in HEK293 cells and their cell-surface expression at steady state was measured by intact cell ligand binding using [^3^H]-RX821002 at 20 nM. Consistent with the remarkable inhibitory effect of mutation of Leu48 on α_2B_-AR cell-surface expression, mutation of Leu64 markedly reduced the cell-surface number of α_2A_-AR by 87%. Surprisingly, in contrast to mutation of Arg49 which did not have significant effects on α_2B_-AR cell-surface expression, mutation of Lys65 to Ala significantly attenuated α_2A_-AR expression at the cell surface by 52%. Double mutation of Leu48/Arg49 in α_2B_-AR and Leu64/Lys65 α_2A_-AR almost abolished their cell-surface transport (Fig. 1C and 1D). To exclude the possibility that these mutations could influence α_2A_-AR binding to the ligand, α_2A_-AR and its mutants were tagged with HA at their N-termini and their cell-surface expression was measured by flow cytometry following staining with anti-HA antibodies in nonpermeabilized cells. The cell-surface expression of the mutants L64A, K65A and LK-AA was reduced by 81, 58 and 93%, respectively, as compared with their wild-type counterpart (Fig. 1E).

To determine if these mutations could alter the total expression of the receptors, α_2A_-AR and α_2B_-AR and their mutants tagged with GFP at their C-termini were transiently expressed in HEK293 cells and their overall expression was determined by flow cytometry measuring the GFP signal. In contrast to the cell-surface expression, these mutations did not significantly alter the overall expression of α_2A_-AR (Fig. 1C) and α_2B_-AR (Fig. 1D). These data, together our previous data [38], demonstrate that the single Leu residue in the ICL1 plays a general role in the cell-surface transport of GPCRs, whereas its neighboring positively charged residue may differentially regulate the cell-surface targeting of α_2A_-AR and α_2B_-AR.

### Intracellular Accumulation of α_2A_-AR Induced by Mutation of Leu64 and Lys65

**Figure 2:**
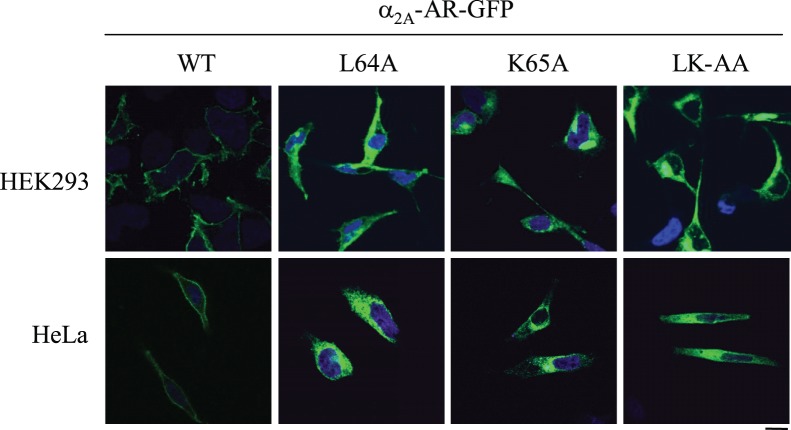
Effects of mutating Leu64 and Lys65 residues on the subcellular distribution of α_2A_-AR. GFP-tagged wild-type (WT) α_2A_-AR and its mutants L64A, K65A and LK-AA were transiently expressed in HEK293 (*upper panel*) and HeLa cells (*lower panel*) and their subcellular distribution of the receptors was revealed by detecting GFP fluorescence by confocal microscopy. The data shown are representative images of at least three independent experiments. *Green*, GFP-tagged receptors; *blue*, DNA staining by DAPI (nuclei). Scale bar, 10 µm.

To further confirm the inhibitory effect of mutation of Leu64 and Lys65 on the cell-surface transport of α_2A_-AR, the subcellular distribution of GFP-tagged α_2A_-AR and its mutants in HEK293 cells was visualized by confocal microscopy. As expected, wild-type α_2A_-AR was robustly expressed at the cell surface. Mutation of Leu64 and Lys65 to Ala individually or in combination caused a remarkable accumulation of α_2A_-AR in the perinuclear region (Fig. 2).

To determine if the effect of the mutations on the subcellular distribution of α_2A_-AR is cell-type specific, GFP-tagged α_2A_-AR and its mutants were transiently expressed in HeLa cells. Similar to the results obtained in HEK293 cells, the α_2A_-AR mutants L64A, K65A and LK-AA displayed a strong intracellular distribution pattern in HeLa cells (Fig. 2). These data suggest that both Leu64 and Lys65 are able to regulate α_2A_-AR cell-surface expression in different cell types.

### Effect of Mutation of Lys65 to Arg, Glu and Gln on the Cell-surface Expression and Subcellular Distribution of α_2A_-AR

**Figure 3:**
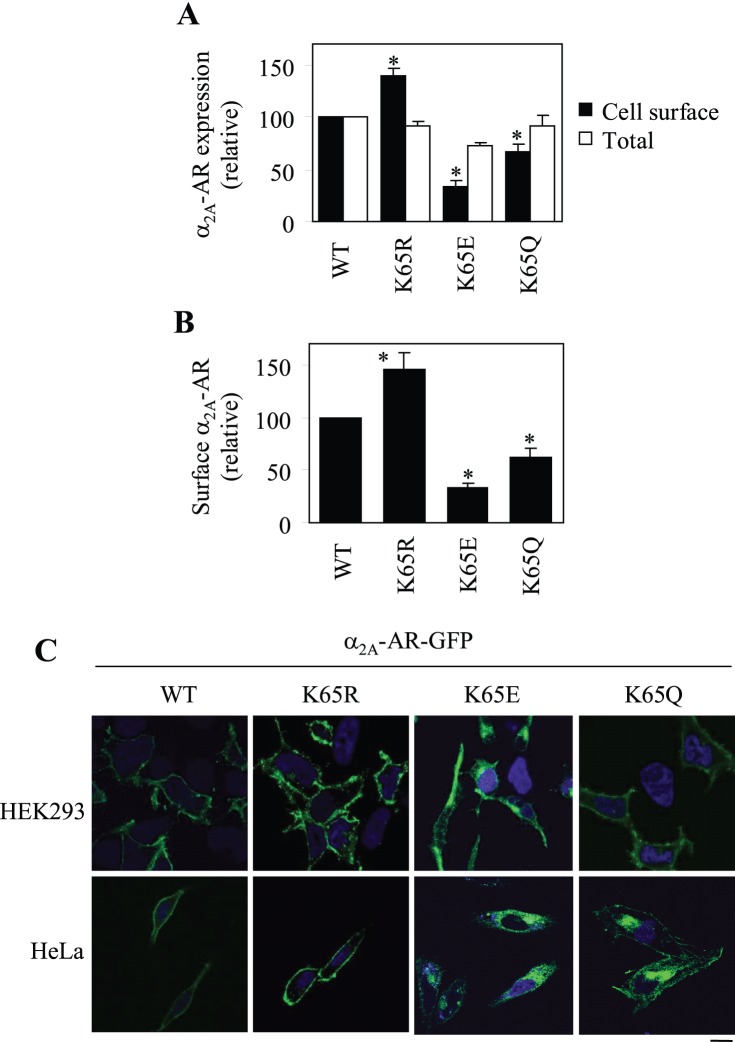
Effects of mutating Lys65 to Arg, Glu and Gln on the cell-surface expression and subcellular distribution of α_2A_-AR. (A) Quantification of the cell surface and total expression of α_2A_-AR and its Lys mutants. HEK293 cells were transfected with α_2A_-AR and its mutants. The cell-surface expression of the receptors was measured by intact cell binding assays using [^3^H]-RX821002 and total receptor expression by flow cytometry measuring the GFP signal as described in the legends of figure 1. (B) Quantification of the cell-surface expression of α_2A_-AR and its mutants by flow cytometry following staining with anti-HA antibodies in nonpermeabilized cells as described in the legends of figure 1. The data shown in (A) and (B) are percentages of the mean value obtained from cells transfected with wild-type (WT) α_2A_-AR and are presented as the mean ± S.E. of four experiments. *, *p*<0.05 *versus* WT α_2A_-AR. (C) Effect of mutation of Lys65 on the subcellular distribution of α_2A_-AR. α_2A_-AR and its mutants K65R, K65E and K65Q were tagged with GFP at their C-termini and transiently expressed in HEK293 (*upper panel*) and HeLa cells (*lower panel*). Their subcellular distribution was revealed by detecting GFP fluorescence by confocal microscopy. The data shown are representative images of at least three independent experiments. *Green*, GFP-tagged receptors; *blue*, DNA staining by DAPI (nuclei). Scale bar, 10 µm.

Our preceding data have revealed that Lys65 plays an important role in modulating α_2A_-AR cell-surface expression. To define the possible underlying molecular mechanisms, we first determined the role of its positively charged property. Lys65 was mutated to the same charged Arg residue, opposite charged Glu residue and non-charged Gln residue and the effects of these mutations on α_2A_-AR expression at the cell surface were defined by intact cell ligand binding and subcellular distribution analysis. Similar to its Ala mutation, mutation of Lys65 to Glu and Gln inhibited α_2A_-AR expression at the cell surface by 67 and 36%, respectively, as measured by intact cell ligand binding (Fig. 3A). More interestingly, mutation of Lys65 to Arg significantly augmented the cell-surface expression of α_2A_-AR by 42% (Fig. 3A). Similar results were obtained by flow cytometry to measure the cell-surface expression of HA-tagged α_2A_-AR and its mutants K65R, K65E and K65Q (Fig. 3B).

Consistent with the measurement of the cell-surface expression by ligand binding and flow cytometry, analysis of the subcellular distribution of the receptors showed that wild-type α_2A_-AR and its mutant K65R strongly expressed at the cell surface, whereas the mutants K65E and K65Q were largely accumulated inside the cell, unable to transport to the cell surface in both HEK293 and HeLa cells (Fig. 3C).

### Functional Regulation of α_2A_-AR by Mutation of Lys65

**Figure 4:**
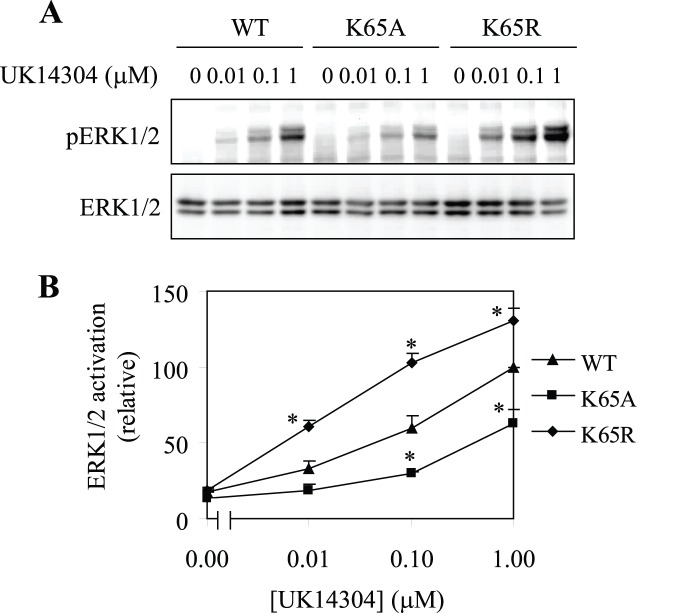
Effects of mutating Lys65 to Ala and Arg on α_2A_-AR-mediated activation of ERK1/2. (A) HEK293 cells were transfected with wild-type (WT) α_2A_-AR or its mutants K65A and K65R and then stimulated with increasing concentrations of UK14,304 for 5 min. ERK1/2 activation was determined by Western blot analysis using phospho-specific ERK1/2 antibodies. *Upper panel*, a representative blot of ERK1/2 activation; *Lower panel*, total ERK1/2 expression. (B) Quantitative data expressed as percentage of ERK1/2 activation obtained in cells transfected with α_2A_-AR and stimulated with UK14304 at 1 µM and presented as the mean ± S.E. of three separate experiments. *, *p*<0.05 *versus* WT α_2A_-AR at the same concentration of UK14,304.

We then sought to determine if the mutation of Lys65 could alter the function of α_2A_-AR by using the agonist-mediated activation of ERK/12 as readout. HEK293 cells were transiently transfected with α_2A_-AR and its mutants K65R and K65A and their abilities to activate ERK1/2 in response to stimulation with the α_2_-AR agonist UK14,304 were compared. ERK1/2 were activated by UK14,304 in a dose-dependent fashion in cells expressing α_2A_-AR and the dose-dependent activation of ERK1/2 was clearly inhibited in cells transfected with the mutant K65A (Fig. 4A and 4B). In contrast, ERK1/2 activation by UK14,304 was significantly higher in cells expressing the mutant K65R than in cells expressing wild-type α_2A_-AR (Fig. 4A and 4B). These data are consistent with the opposing effects of Lys65 mutation to Ala and Arg on the cell-surface expression of α_2A_-AR. These data also suggest that Lys65 in the ICL1 modulates not only receptor trafficking but also receptor signaling.

### Lys65 Likely Modulates α_2A_-AR Transport at the ER

**Figure 5:**
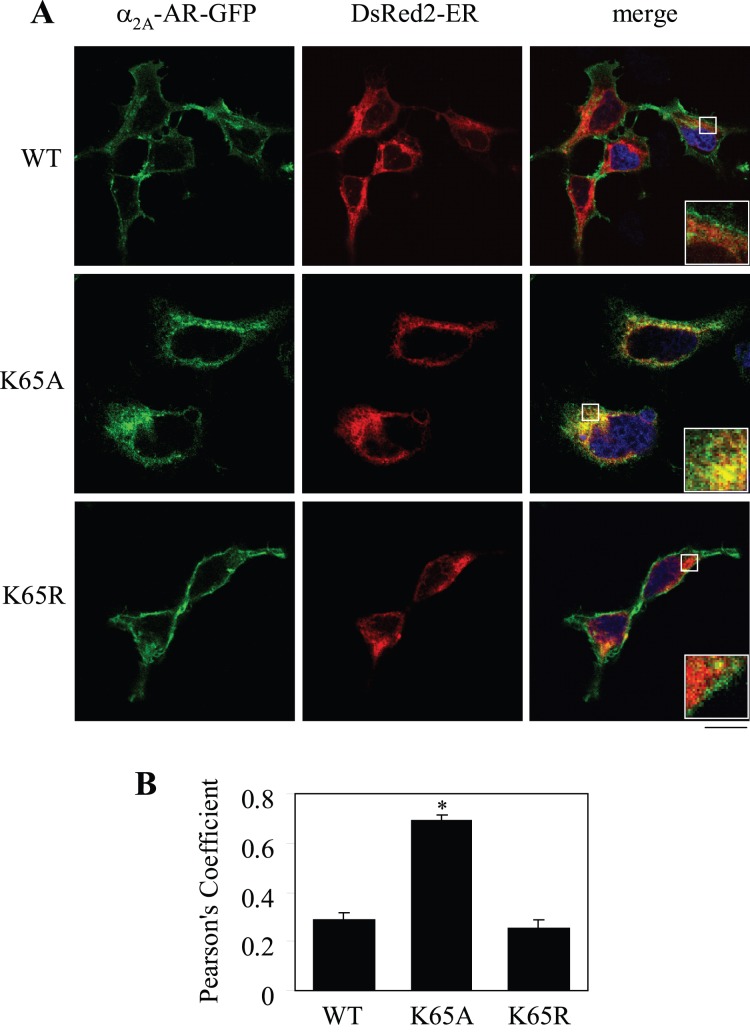
Effect of mutation of Lys65 on the colocalization of α_2A_-AR with the ER marker DsRed2-ER. (A) HEK293 cells were transiently transfected with the GFP-tagged α_2A_-AR or its Lys65 mutants together with pDsRed2-ER. The subcellular distribution and co-localization of the receptors with the ER marker DsRed2-ER were revealed by confocal fluorescence microscopy as described under “Materials and Methods”. *Green*, α_2A_-AR or its mutants tagged with GFP; *red*, DsRed2-ER; *yellow*, co-localization of α_2A_-AR or its mutants with the ER marker DsRed2-ER; *blue*, DNA staining by DAPI (nuclei). The data shown are representative images of at least three independent experiments. (B) Quantification of Pearson’s coefficient between the receptors and the ER marker. The data are presented as the mean ± S.E. of 20 cells from three different experiments. *, *p*<0.05 *versus* WT α_2A_-AR. Scale bar, 10 µm.

To define the intracellular compartment where the residue Lys65 influences α_2A_-AR transport, GFP-tagged α_2A_-AR and its mutants K65A and K65R were co-localized with different intracellular markers. The mutant K65A was extensively co-localized with the ER marker DsRed2-ER (Fig. 5A), but not the Golgi marker GM130 (data not shown). In contrast, wild-type α_2A_-AR and its mutant K65R did not clearly co-localize with DeRed2-ER (Fig. 5A). To quantify the colocalization of the receptors with the ER marker, Pearson’s coefficient was determined. Pearson’s coefficient of the mutant K65A was significantly higher than those of wild-type α_2A_-AR and the mutant K65R (Fig. 5B). These data suggest that the residue Lys65 in the ICL1 likely controls the exit of α_2A_-AR out of the ER.

## Discussion

The molecular mechanisms underlying export from the ER and subsequent transport to the cell surface of the GPCR superfamily remain poorly elucidated. It has been significant efforts to define the structural determinants for GPCR export and several highly conserved hydrophobic and charged sequences which are required for GPCR export from the ER to the cell surface have been identified [5], [15], [18], [24]–[34], [37], [38], [49]. We have previously investigated the role of the ICL1 in the cell-surface transport of GPCRs and identified a Leu residue essential for ER export of a group of GPCRs, including α_2B_-AR [38]. This Leu residue is extremely conserved amongst the family A GPCRs and indeed, as demonstrated in this manuscript, Leu64 in the ICL1 also plays an obligatory role in α_2A_-AR export from the ER and transport to the cell surface. Thus, this isolated Leu may provide a common signal directing anterograde transport of multiple nascent GPCRs.

The most important finding described in this manuscript is that different positively charged residues on the ICL1 have differential impacts on the cell-surface transport of distinct GPCRs. During the studies on the function of the ICL1, we have surprisingly found that mutation of Lys65 produced a marked inhibitory effect on the cell-surface transport of α_2A_-AR. First, intact cell ligand binding and flow cytometry to quantify the cell-surface receptors showed that mutation of Lys65 to Ala reduced α_2A_-AR cell-surface number by more than 50%. Second, the significant reduction of receptor expression at the cell surface was supported by direct visualization of intracellular localization of the mutated receptors. As revealed by confocal microscopy, GFP-tagged K65A was extensively expressed inside the cell which is in contrast to robust cell-surface expression of its wild-type counterpart. Third, the function of Lys65 in controlling α_2A_-AR transport is also supported by receptor-mediated signal propagation as measured by ERK1/2 activation. The reduction of ERK1/2 activation in cells expressing K65A as compared with cells expressing wild-type α_2A_-AR could be simply due to less expression of the mutated α_2A_-AR at the cell surface, which are available for binding to the ligand, indicating that mutation of Lys65 not only reduced the cell-surface expression of α_2A_-AR but also attenuated receptor-mediated signal propagation. These data strongly indicate that, in addition to Leu64, Lys65 in the ICL1 also plays a crucial role in regulating cell-surface transport of α_2A_-AR. By contrast, mutation of the positively charged residue at the same position, Arg49, did not influence the cell-surface transport of α_2B_-AR. These data demonstrated for the first time that a single positively charged residue on the short ICL1 is involved in the regulation of export trafficking of GPCRs in a receptor subtype-specific fashion.

It is likely that Lys65 residue modulates α_2A_-AR transport at the level of the ER. We found that the α_2A_-AR mutant K65A was extensively co-localized with the ER marker DsRed2-ER, suggesting that the mutant was unable to exit from the ER where they are synthesized. Therefore, these studies provide another novel regulatory mechanism for the ER export of nascent α_2A_-AR. As this single Lys residue also exists in several other group A GPCRs, including angiotensin II, muscarinic and chemokine receptors [38] (data not shown), it may function as an important code which not only directs the ER export but also controls the cell-surface availability of these GPCRs. These data, together with our previous studies identifying the F(x)_6_LL, RRR and YS motifs [15], [34], [37], [40], have strongly demonstrated that export trafficking of α_2_-AR is coordinated by many structural determinants and export motifs located in the intracellular domains of the receptors.

The function of Lys65 residue in regulating the ER export and cell-surface expression of α_2A_-AR is likely dictated by its specific physiochemical and structural features. First, our data demonstrating that mutation of Lys65 to Ala, Glu and Gln significantly inhibited α_2A_-AR export from the ER and transport to the cell surface suggest that the positively charged property is an important factor determining the function of Lys65 in α_2A_-AR export. It is possible that Lys65 plays a role in the correct folding or proper assembly of α_2A_-AR in the ER and thus, its mutation results in the misfolding and defective export of the receptor. It is also possible that Lys65 may mediate α_2A_-AR interaction with other regulatory proteins and such interactions are crucial for the receptor export from the ER. Consistent with this possibility, a number of accessory proteins directly interact with the intracellular loops of GPCRs to modulate receptor export from the ER to the cell surface [5]. This possibility is also supported by our recent studies showing that the RRR motif in the ICL3 mediates α_2B_-AR interaction with Sec24 isoforms, components of COPII transport vesicles, to control receptor export from the ER [34].

Second, more interestingly, mutation of Lys65 to the same charged Arg residue enhanced the cell-surface number of α_2A_-AR. There are at least two possible explanations for the enhancement of the cell-surface expression of α_2A_-AR induced by mutation of Lys65 to Arg. It is possible that mutation of Lys to Arg stabilizes α_2A_-AR once transported to the cell surface. As the overall size of the side chain of Arg is larger than that of Lys, it may enhance the possible ionic interactions between the positively charged side chain and some negatively charged components embedded within the plasma membrane. It is also possible that mutation of Lys to Arg may reduce the targeting of the cell-surface α_2A_-AR to some degradation pathways, such as those mediated by ubiquitination and/or sumoylation which are carried out specifically on Lys residues. However, whether or not Lys65 residue indeed undergoes ubiquitination and/or sumoylation remains unknown. Nevertheless, this study has demonstrated a crucial role for Lys65 residue in regulating the ER export and cell-surface expression of α_2A_-AR, yet the precise mechanisms of its actions remain to be elucidated.

It has become increasingly clear that the efficient trafficking and precise positioning to specific functional destination of GPCRs are critical aspects in controlling integrated responses of the cell to hormones. Indeed, defective anterograde transport of GPCRs to the cell surface *en route* from the ER through the Golgi is tightly associated with the pathogenesis of a variety of human diseases, including those induced by naturally occurring mutations or truncations of GPCRs, leading to the accumulation of misfolded receptors in the ER [16], [50], [51]. Therefore, further elucidation of the molecular mechanisms underlying the export traffic of GPCRs may provide a foundation for development of therapeutic strategies by designing specific drugs to control GPCR biosynthesis and cell-surface export trafficking.
